# Supplementary material for: Combination of plasma MMPs and PD-1-binding soluble PD-L1 predicts recurrence in gastric cancer and the efficacy of immune checkpoint inhibitors in non-small cell lung cancer
Source: Front Pharmacol. 2024 May 7;15:1384731. doi: 10.3389/fphar.2024.1384731 (PMC11106465; doi:10.3389/fphar.2024.1384731)
Supplement: Supplementary file 1 [file Image5.pdf]

Supplementary Figure 5

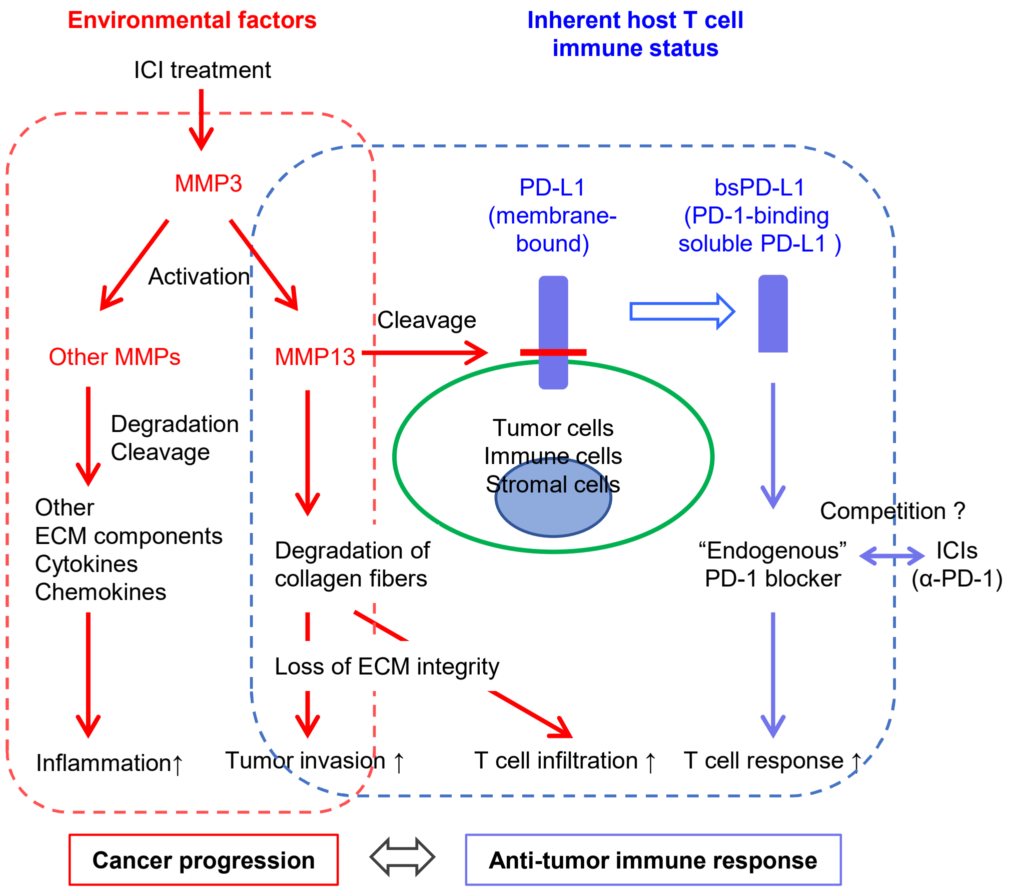

Schematic model of the regulation of the tumor microenvironment by bsPD-L1 and MMPs in NSCLC patients with ICI treatment. Baseline levels of bsPD-L1 or MMP13 indicate host inherent T cell immune status prior to ICI treatment. MMP13 cleaves membrane-bound PD-L1, resulting in bsPD-L1 production. bsPD-L1 may compete with ICIs such as anti-PD-1 to bind to PD-1. MMP13 and its activator MMP3 levels change during ICI treatment. MMP3 activates a wide range of MMPs and degrades a broad range of extracellular matrix (ECM) and non-matrix substrates, including cytokines and chemokines. The increase in MMP3 and MMP13 induces inflammation and accelerates cancer progression rather than T cell response. The host inherent T cell immune status and the change of MMPs (sensitivity to ICIs) vary among patients.
