# Supplementary material for: Combination of plasma MMPs and PD-1-binding soluble PD-L1 predicts recurrence in gastric cancer and the efficacy of immune checkpoint inhibitors in non-small cell lung cancer
Source: Front Pharmacol. 2024 May 7;15:1384731. doi: 10.3389/fphar.2024.1384731 (PMC11106465; doi:10.3389/fphar.2024.1384731)
Supplement: Supplementary file 3 [file Table2.pdf]

Supplementary Table 2 Characteristics of NSCLC patients

**Variable**		All patients (N=72)	bsPD-L1-negative (N=56)	bsPD-L1-positive (N=16)	p value
Age	Median (range)	67 (28-88)	68.5 (28-82)	68.5 (43-88)	0.3312
Gender					0.3247
	Male	56(77.8%)	45(80.4%)	11(68.8%)	
	Female	16(22.2%)	11(19.6%)	5(22.2%)	
Smoking					**0.0441**
	Never	11(15.3%)	6(10.7%)	5(31.2%)	
	Current or Former	61(84.7%)	50(89.3%)	11(68.8%)	
EGFR Mutation					0.4876
	Wild	60(88.2%)	46(86.8%)	14(93.3%)	
	Mutant	8(11.8%)	7(13.2%)	1(6.7%)	
T					0.5127
	-1c	18(25.0%)	13(23.2%)	5(31.3%)	
	2a-	54(75.0%)	43(76.8%)	11(68.7%)	
N					0.2551
	0	11(15.3%)	10(19.6%)	1(6.3%)	
	1 -	61(84.7%)	46(80.3%)	15(93.7%)	
M					0.9461
	0	49(68.1%)	38(67.9%)	11(68.7%)	
	1	23(31.9%)	18(32.1%)	5(31.3%)	
Disease stage					0.3164
	I - II	10 (13.9%)	9 (16.1%)	1 (6.3%)	
	III - IV	62(86.1%)	47 (83.9%)	15 (93.8%)	
Histology					0.7473
	Squamous cell carcinoma	32 (44.4%)	24 (42.9%)	8 (50.0%)	
	Adenocarcinoma	32 (44.4%)	25 (44.6%)	7 (43.8%)	
	Non-small	8 (11.2%)	7 (12.5%)	1 (6.2%)	
PD-L1 TPS status					0.1724
	<50	31 (52.5%)	22 (47.8%)	9 (69.2%)	
	50≤	28 (47.5%)	24 (52.2%)	4 (30.8%)	
