# Supplementary material for: Combination of plasma MMPs and PD-1-binding soluble PD-L1 predicts recurrence in gastric cancer and the efficacy of immune checkpoint inhibitors in non-small cell lung cancer
Source: Front Pharmacol. 2024 May 7;15:1384731. doi: 10.3389/fphar.2024.1384731 (PMC11106465; doi:10.3389/fphar.2024.1384731)
Supplement: Supplementary file 4 [file Image4.pdf]

Supplementary Figure 4

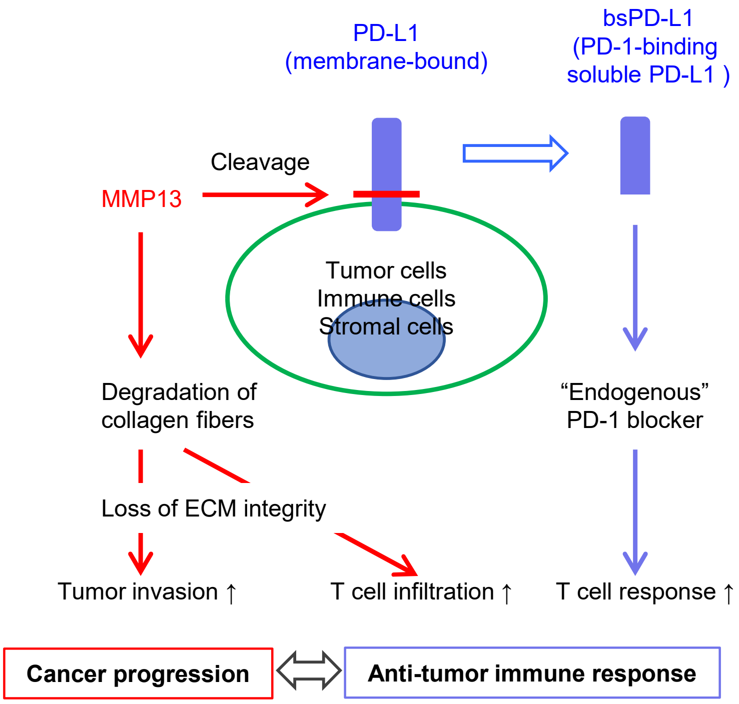

Schematic model of the regulation of the tumor microenvironment by bsPD-L1 and MMP13 in GC patients. MMP13 cleaves membrane-bound PD-L1 to generate bsPD-L1. bsPD-L1 binds to PD-1 and functions as an endogenous PD-1 blocker. MMP13 degrades collagen fibers in tumor tissue, resulting in loss of extracellular matrix (ECM) integrity, which causes tumor invasion as well as T cell infiltration. The balance between the anti-tumor immune response and cancer progression is important for prognosis of GC patients. High levels of MMP13 promotes tumor invasion rather than T cell response, leading to poor prognosis.
