# Supplementary material for: Combination of plasma MMPs and PD-1-binding soluble PD-L1 predicts recurrence in gastric cancer and the efficacy of immune checkpoint inhibitors in non-small cell lung cancer
Source: Front Pharmacol. 2024 May 7;15:1384731. doi: 10.3389/fphar.2024.1384731 (PMC11106465; doi:10.3389/fphar.2024.1384731)
Supplement: Supplementary file 5 [file Image2.pdf]

Supplementary Figure 2

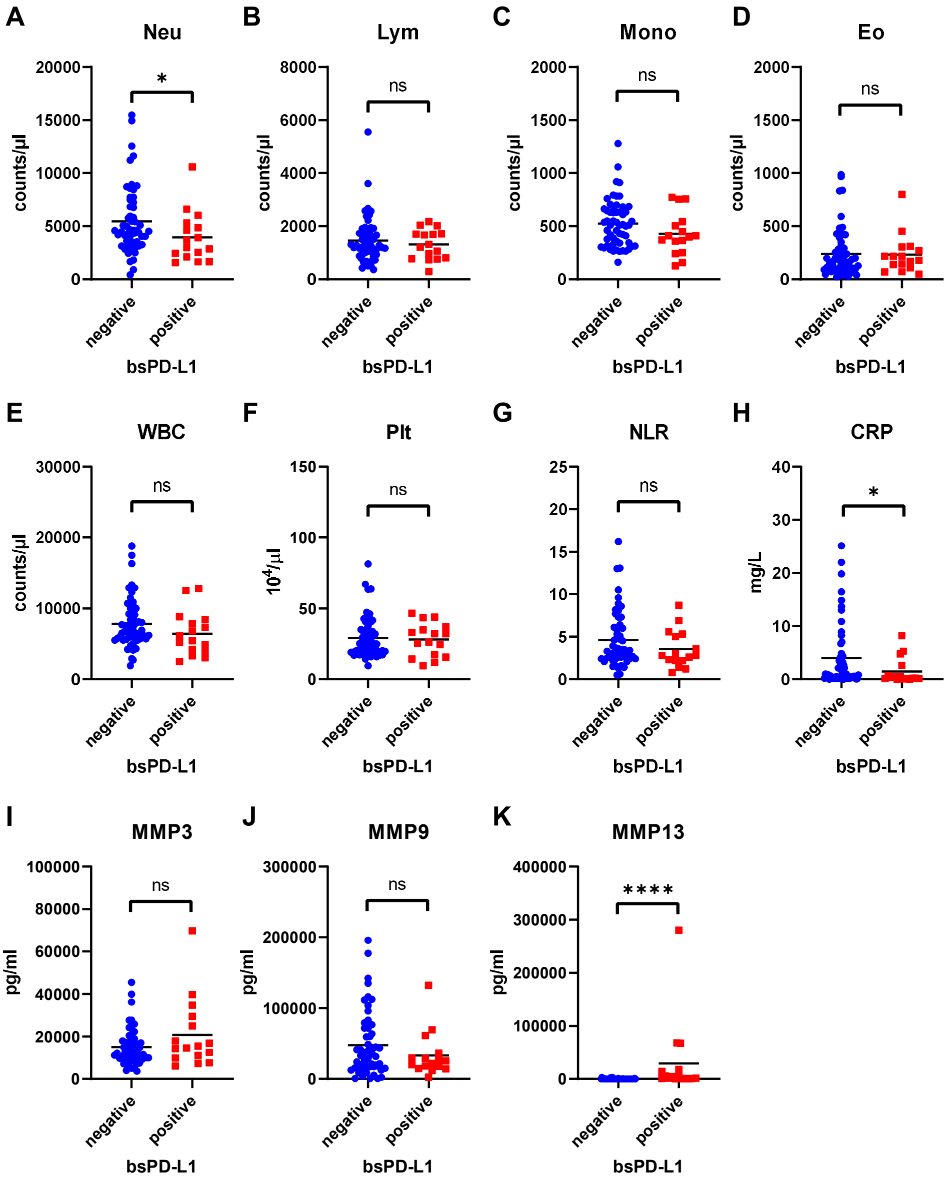

Comparison of inflammatory markers between bsPD-L1$^+$ and bsPD-L1$^-$ NSCLC patients. Counts of (A) Neutrophil, (B) lymphocyte, (C) monocyte, (D) eosinophil, (E) white blood cell, and (F) platelet, (G) neutrophil-to-lymphocyte ratio, and levels of (H) C-reactive protein, and (I–K) MMPs in bsPD-L1$^+$ (n = 16) and bsPD-L1$^-$ (n = 56) NSCLC patients. The horizontal lines indicate the mean. Statistical significance was calculated using the Student's t-test (A, B, and E) or the Mann–Whitney U test (C, D, F, G, H, I, J, and K). $*p < 0.05$; $****p < 0.0001$; ns, not significant.
