# Supplementary material for: Combination of plasma MMPs and PD-1-binding soluble PD-L1 predicts recurrence in gastric cancer and the efficacy of immune checkpoint inhibitors in non-small cell lung cancer
Source: Front Pharmacol. 2024 May 7;15:1384731. doi: 10.3389/fphar.2024.1384731 (PMC11106465; doi:10.3389/fphar.2024.1384731)
Supplement: Supplementary file 6 [file Image3.pdf]

Supplementary Figure 3

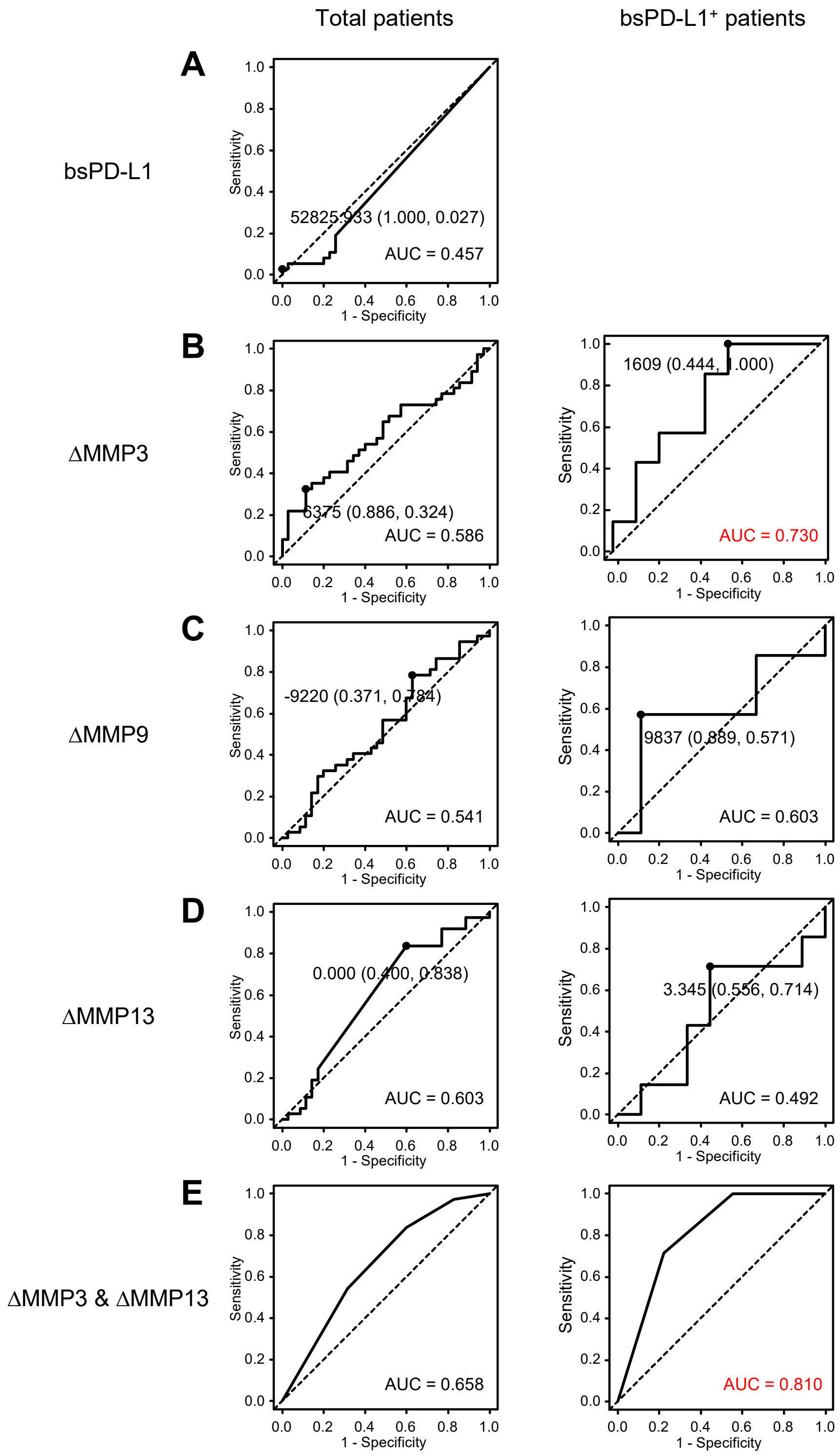

ROC curves for OS in NSCLC patients. (A) ROC curves of bsPD-L1 in total patients (n = 72). (B) ROC curves of MMP3 change in total (n=72) and bsPD-L1^{+} (n = 16) patients. (C) ROC curves of MMP9 change in total (n=72) and bsPD-L1^{+} (n = 16) patients. (D) ROC curves of MMP13 change in total (n=72) and bsPD-L1^{+} (n = 16) patients. (E) Multivariate ROC curves of combined MMP3 and MMP13 changes in total (n=72) and bsPD-L1^{+} (n = 16) patients.
