# Supplementary material for: Combination of plasma MMPs and PD-1-binding soluble PD-L1 predicts recurrence in gastric cancer and the efficacy of immune checkpoint inhibitors in non-small cell lung cancer
Source: Front Pharmacol. 2024 May 7;15:1384731. doi: 10.3389/fphar.2024.1384731 (PMC11106465; doi:10.3389/fphar.2024.1384731)
Supplement: Supplementary file 7 [file Image1.pdf]

Supplementary Figure 1

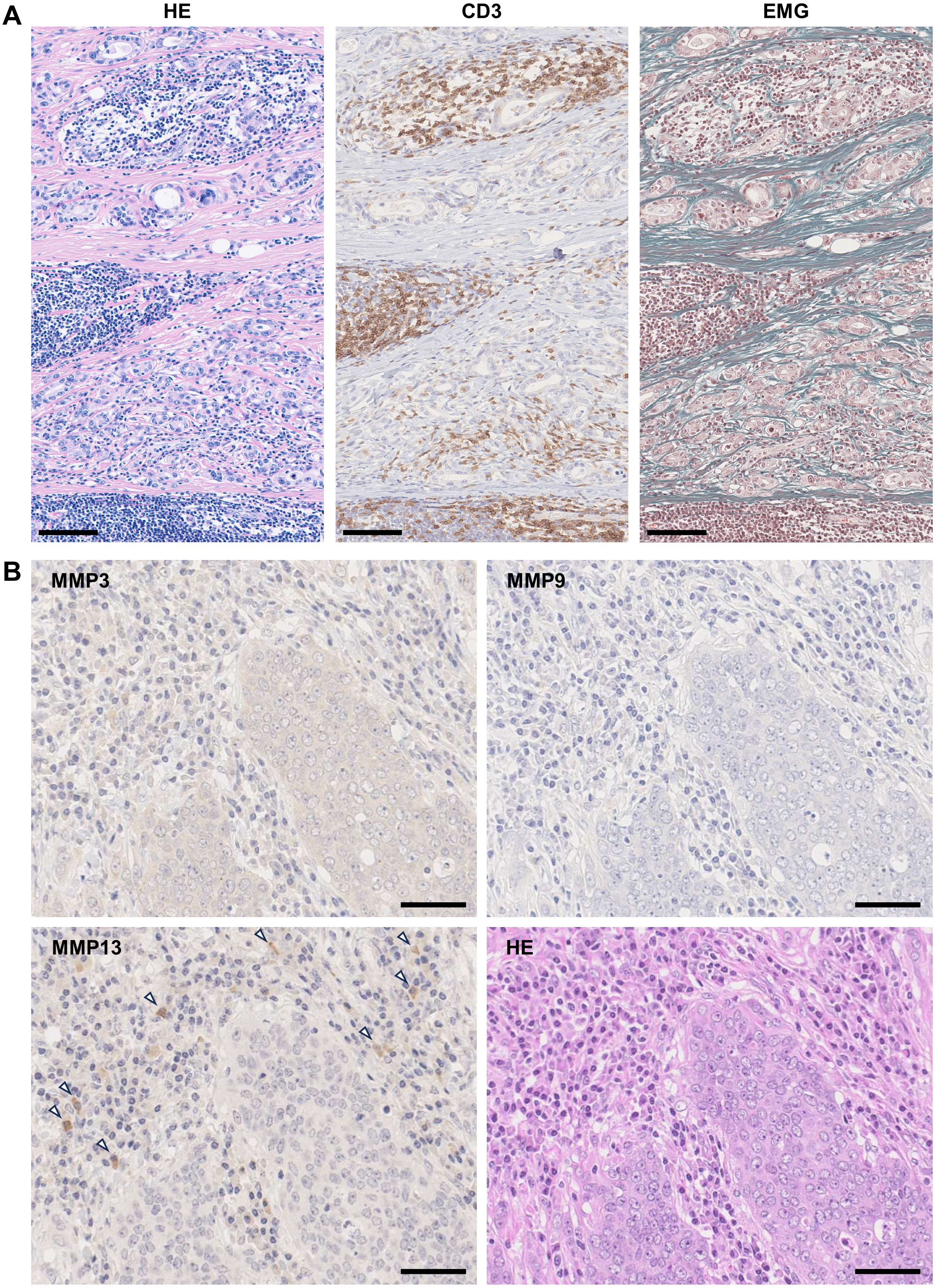

Histological analysis of gastric cancer tissues. (A) Serial sections of tumor tissue from Patient 86 were analyzed by H&E, anti-CD3, and EMG staining. Original magnification, 20×. Scale bars indicate 100 µm. (B) Representative images of tumor tissues from bsPD-L1^{+} patients. Serial sections were analyzed by anti-MMP3, 9, and 13 and H&E staining. Original magnification, 20×. Scale bars indicate 50 µm. Nuclei were counterstained with hematoxylin (blue). The arrow heads represent MMP13^{+} cells.
